# The clinical benefit of pegylated liposomal doxorubicin in patients with metastatic breast cancer previously treated with conventional anthracyclines: a multicentre phase II trial

**DOI:** 10.1038/sj.bjc.6603158

**Published:** 2006-05-09

**Authors:** S-E Al-Batran, J Bischoff, G von Minckwitz, A Atmaca, U Kleeberg, I Meuthen, G Morack, W Lerbs, D Hecker, J Sehouli, A Knuth, E Jager

**Affiliations:** 1 Department of Hematology and Oncology, Krankenhaus Nordwest, 60488 Frankfurt am Main, Frankfurt, Germany; 2 Klinik Bad Trissl, Oberaudorf, Germany; 3 Universitats-Frauenklinik/German BreastGroup, Frankfurt, Germany; 4 Hämatologisch-Onkologische Praxis Altona, Hamburg, Germany; 5 Krankenhaus Holweide, Köln, Germany; 6 Klinikum Berlin-Buch, Berlin, Germany; 7 Essex pharma, München, Germany; 8 Frauenklinik Charité Campus Mitte, Berlin, Germany; 9 Universitätsspital Zürich, Zürich, Switzerland

**Keywords:** liposomal doxorubicin, breast cancer, anthracycline pretreated

## Abstract

This study evaluates the clinical benefit of pegylated liposomal doxorubicin (PLD) in patients with metastatic breast cancer (MBC), previously treated with conventional anthracyclines. Seventy-nine women with MBC previously treated with anthracyclines received PLD 50 mg m^−2^ every 4 weeks. All patients were previously treated with chemotherapy and 30% of patients had ⩾3 prior chemotherapies for metastatic disease. Patients were considered anthracycline resistant when they had disease progression on anthracycline therapy for MBC or within 6 months of adjuvant therapy. The overall clinical benefit rate (objective response+stable disease ⩾24 weeks) was 24% (16.1% in patients with documented anthracycline resistance *vs* 29% in patients classified as having non-anthracycline-resistant disease). There was no difference with respect to the clinical benefit between patients who received PLD >12 months and those who received PLD ⩽12 months since last anthracycline treatment for metastatic disease (clinical benefit 25 *vs* 24.1%, respectively). Median time to progression and overall survival were 3.6 and 12.3 months, respectively. The median duration of response was 12 months, and the median time to progression in patients with stable disease (any) was 9.5 months. Fourteen patients (17.7%) had a prolonged clinical benefit lasting ⩾12 months. In conclusion, PLD was associated with an evident clinical benefit in anthracycline-pretreated patients with MBC.

The treatment of patients with metastatic breast cancer (MBC), who failed one or more previous chemotherapy regimens, and whose favourable performance status justifies further treatment, represents a significant challenge to the oncologist. In patients with MBC, anthracyclines and taxanes are the most active drugs. However, the majority of pretreated patients with MBC had been exposed to anthracycline- and taxane-based therapies either in the adjuvant or in the metastatic setting. Here, the repeated use of conventional anthracyclines is limited by cumulative cardiac toxicity and myelosuppression, despite long anthracycline-free intervals in many patients (Singal and Iliskovic, 1998; Pai and Nahata, 2000). In these cases, pegylated liposomal doxorubicin (PLD) may represent an attractive option. Pegylated liposomal doxorubicin has demonstrated comparable efficacy to doxorubicin with a favourable toxicity profile (Gabizon and Martin, 1997; Ranson *et al*, 1997; Lyass *et al*, 2000; Symon *et al*, 2000; Gabizon *et al*, 2003; O'Shaughnessy, 2003; Theodoulou and Hudis, 2004). It was associated with less alopecia, myelotoxicity, and cardiac toxicity than free doxorubicin but higher rates of palmar–plantar erythrodysesthesia (PPE) and mucositis (O'Brien *et al*, 2004). Preclinical and clinical studies have indicated that PLD may not be completely cross-resistant with conventional anthracyclines (Gabizon *et al*, 2003; O'Brien *et al*, 2004). Based on these results, we believe that there is a rational basis for the use of PLD as a second-, third-, or fourth-line therapy in patients with MBC after anthracycline and/or taxane failure. However, in fact, there have been no clinical studies evaluating its clinical benefit in this setting. The present study evaluates the clinical benefit of PLD in an anthracycline-pretreated population and attempts to identify the patients who are more likely to benefit from the treatment.

## PATIENTS AND METHODS

### Patient eligibility

Female patients were eligible with histologically confirmed MBC with at least one measurable lesion, at least one prior chemotherapy for metastatic disease, Karnofsky performance status ⩾70%, age >18 years, life expectancy ⩾3 months, no concurrent uncontrolled medical illness, no other malignancies (with the exception of squamous cell carcinoma of the skin treated by surgery), baseline left ventricular ejection fraction (LVEF) >50%, and sufficient hepatic and bone marrow function. Number, type, and cumulative dose of previous chemotherapy were not limited. Patients were excluded from the study if they had cardiac diseases including congestive heart failure, atrial or ventricular arrhythmia, were pregnant or breast-feeding. Women with child-bearing potential were advised to take adequate precautions to prevent pregnancy. Participants gave written informed consent before they entered the study, which was approved by the Ethics Committees responsible for the participating centers.

### Chemotherapy

The initial infusion duration of PLD 50 mg m^−2^ was 1 h in order to minimise the risk of infusion-related reactions. Subsequent doses were given as a 30-min infusion. Cycles were repeated every 4 weeks and treatment was continued until disease progression, unacceptable toxicity, patient's refusal, or physician's decision. Antiemetic prophylaxis was given according to local protocols. All patients received vitamin B_6_ (Pyridoxine) 300 mg orally once daily after breakfast during the treatment to prevent PPE (Nagore *et al*, 2000).

### Toxicity assessment

Toxicity was graded according to the National Cancer Institute Common Toxicity Criteria (NCI-CTC) version 2. Cardiac toxicity was based on echocardiographic LVEF measurements and a 12-lead electrocardiogram that were performed at baseline and every 8 weeks. Dose modifications of PLD were permitted for haematological toxicity, increases in total bilirubin, cardiac toxicity, PPE, mucositis, and other NCI-CTC grade 3 or 4 events.

### Assessment of response

Responses were classified according to World Health Organization (WHO) criteria. Computed tomography (CT) scans of measurable lesions were carried out within 4 weeks before the start of the treatment and were repeated every two cycles. Responses were to be confirmed by subsequent CT scans 4–8 weeks after the initial response documentation. Patients who discontinued the study were evaluated at least every 3 months. Patients were considered assessable for response if they had early disease progression or had received at least two cycles of treatment with at least one tumor assessment.

### Definitions and statistical analyses

The primary end point of the study was clinical benefit defined as the rate of CR+PR+SD⩾6 months duration. The treatment was considered active if the clinical benefit rate exceeded 33%. Conversely, the treatment was considered inactive if the clinical benefit rate was below 20%. Based on this hypothesis, the inclusion of 100 patients was planned and considered appropriate to generate robust results. Response and toxicity were analysed descriptively. The 95% confidence interval (CI) for response and clinical benefit was calculated. The progression-free survival (PFS) was measured from the start of the treatment until progression or death of any cause. The overall survival (OS) was measured from the start of the treatment until death of any cause. Anthracycline resistance was defined as having disease progression on anthracycline-based therapy for MBC or within 6 months of adjuvant anthracycline-based therapy.

## RESULTS

Between May 2000 and October 2001, 100 female patients with MBC were enrolled in the study at 25 German centres. First patient was included on 25 May 2000 and last patient on 15 October 2001. Seventy-nine of 100 patients were previously treated with anthracyclines. In order to create a more homogeneous patient population, the analysis was confined to the group of patients previously treated with anthracyclines (*n*=79). The median age of patients was 58 years (range 35–79 years) and the median Karnofsky performance status was 90 (range 60–100). Most patients (82.6%) had visceral disease. All patients were pretreated with at least one chemotherapy regimen in the metastatic setting and all had received prior anthracycline chemotherapy. Seventy-seven per cent of the patients had received prior anthracyclines for metastatic disease. The anthracycline-free interval was ⩽12 months in 41.8% of the patients. Patient characteristics and treatment history are listed in Tables 1 and 2. All patients were evaluable for safety and efficacy.

### Safety

Seventy-nine patients received a total of 325 treatment cycles. The median number of cycles was 3 (range 1–12 cycles). The median cumulative dose of PLD was 150 mg m^−2^ (range 50–580 mg m^−2^). A dose reduction was required in 12 of 79 patients (15.1%). Nine patients discontinued treatment because of adverse events possibly related to the study treatment: two for PPE, two for stomatitis, one for prolonged haematological toxicity (thrombocytopenia and neutropenia), and one each for skin reactions other than PPE, allergic reaction, circulatory collapse, and bronchopneumonia. Four out of these nine patients received more than two cycles of treatment (three, three, five, and six cycles). No treatment interruptions or discontinuation for cardiac toxicity and no treatment-related deaths were reported.

Main toxicities are listed in Table 3. Neutropenia (17.1%) and leukopenia (14.4%) were the most frequent NCI-CTC grade 3–4 haematological toxicities. Among the non-haematological adverse events, NCI-CTC grade 3–4 stomatitis (10.2%) was the most prominent. National Cancer Institute-CTC grade 3–4 PPE occurred in five patients (6.4%).

Abnormal electrocardiograms during the treatment were documented in four patients who had normal baseline electrocardiograms. Electrocardiographic changes included tachycardia and tachyarrhythmia that were considered related to the progression of underlying disease. A decrease of LVEF ⩾15% from baseline was not observed.

### Tumor response and clinical benefit

One complete response in 79 evaluable patients (1,3%) and nine (11.4%) partial responses were observed, adding to an overall response rate of 12.7% (95% CI 4.7–20.6%). Twenty-two patients (27.8%; 95% CI 17.3–38.4%) had stable disease and 47 patients (59.5%) had progressive disease. Patients with responses or stable diseases of ⩾6 months duration were considered for clinical benefit, resulting in an overall clinical benefit rate of 24% (95% CI 14.6–33.4%). Patients with less exposure to prior chemotherapy regimens tended to have higher rates of clinical benefit (Table 4). In addition, higher rates of clinical benefit were achieved in taxane naive patients (*χ*^2^, *P*=0.024; Table 5). The clinical benefit was 16.1% in patients classified as having anthracycline-resistant disease *vs* 29% in non-anthracycline-resistant patients (*χ*^2^, *P*=0.186; Table 5). There was no significant difference with respect to the clinical benefit between patients who received PLD >12 months and those who received PLD ⩽12 months since last anthracycline treatment for metastatic disease (clinical benefit 25 *vs* 24.1%, respectively). Fourteen (17.7%) patients experienced prolonged responses (response or stable disease) lasting ⩾12 months. Eight (57.1%) of these patients had an anthracycline-free interval >12 months, nine (64.2%) were classified as having non-anthracycline-resistant disease, and seven (50%) were taxane naive, resulting in no remarkable differences when compared with the full patient population.

### PFS and OS

**Figure 1:**
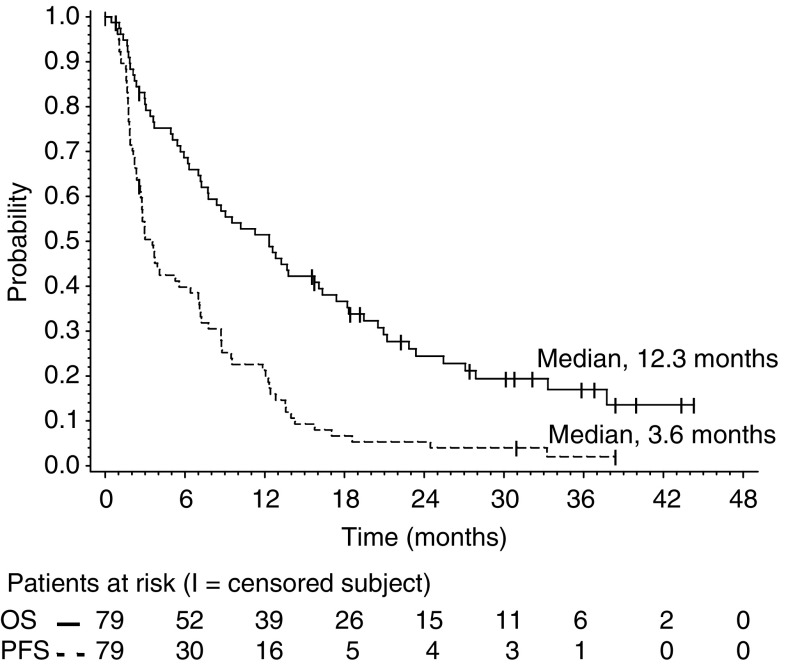
Overall survival and PFS survival for the entire group.

**Figure 2:**
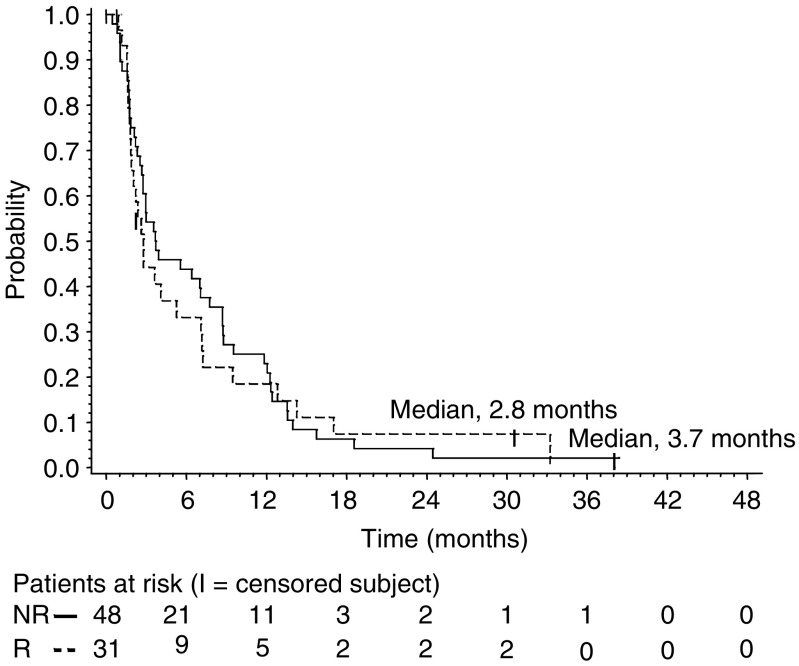
Progression free survival for patients with anthracycline resistance (R) *vs* those considered having anthracycline non-resistant disease (NR).

**Figure 3:**
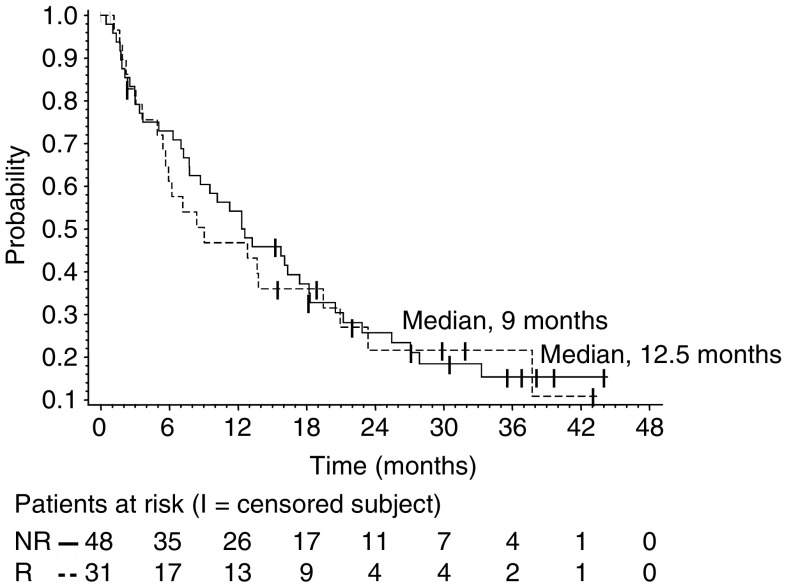
Overall survival for patients with anthracycline resistance (R) *vs* those considered having anthracycline non-resistant disease (NR).

Patients were included in the survival analysis on an intent-to-treat basis. The median follow-up time was 30 months (range 1–47). Median PFS and OS for the entire group were 3.6 months (95% CI 2.7–6.4) and 12.3 months (95% CI 7.7–16.3), respectively. The response duration ranged from 7.0 to 24.5 months (median 12.0 months; 95% CI 8.7–14.0), and the median PFS in patients with stable disease (any) was 9.5 months (95% CI 7.8–13.6). Median PFS and OS were 2.8 (95% CI 1.9–7.1) and 9.0 months (95% CI 5.7–19.5), respectively, for patients classified as having anthracycline-resistant disease *vs* 3.7 (95% CI 2.8–7.8) and 12.5 (95% CI 7.8–18.2) months, respectively, for patients classified as having non-anthracycline-resistant disease. Progression-free survival and OS were assessed by the Kaplan–Meier analysis shown in Figures 1, 2 and 3.

## DISCUSSION

We report here the results of a phase II trial on PLD in patients with MBC previously treated with conventional anthracyclines. The study evaluates the clinical benefit of PLD in patients who had received multiple prior chemotherapies including anthracyclines and taxanes. All the patients analysed in our study had received prior anthracycline therapy, and 68% of the patients had received prior taxane therapy. Moreover, 33% of the patients received the study treatment in the greater than or equal to fourth-line setting, and most had visceral disease. In this patient population, an overall clinical benefit rate of 24% was achieved. The median response duration was 12 months, and the median PFS in patients with stable disease (any) was 9.5 months. The overall clinical benefit observed in our study, must, therefore be considered as modest but evident (Clinical Benefit rate <33% but >20%). Notably, once a response or a stable disease was achieved, it was durable.

The clinical benefit was highest in patients with no prior exposure to taxanes and lowest – but still evident – in patients with documented anthracycline resistance. There was, on the other hand, no significant difference with respect to the clinical benefit between patients who received PLD >12 months and those who received PLD ⩽12 months since last prior anthracycline-based treatment for metastatic disease (clinical benefit 25 *vs* 24.1%, respectively). These findings may indicate that the time that had passed since last anthracycline treatment (>12 or <12 months) may not be a predictor of PLD efficacy, but the fact, whether patient's disease progressed on a prior anthracycline treatment or not. In general, our results are consistent with observations made by Keller *et al* (2004) in a recent phase III study with PLD *vs* vinorelbine in patients with taxane-refractory advanced breast cancer (response rate, median PFS, and OS, 12.7%, 3.6 months, and 12.3 months, respectively, as compared with 10%, 2.9 months, and 11 months, respectively). In this study, PLD was superior to vinorelbine in patients with taxane-refractory MBC who had no prior exposure to an anthracycline and as effective as vinorelbine in patients with anthracycline-resistant disease, demonstrating activity in this group of patients. In this study, however, in contrast to our results, there were no differences in PFS and OS between patients with prior anthracycline exposure and patients with documented anthracycline resistance (median PFS, 2.4 *vs* 2.6 months and median OS, 9.2 *vs* 8.0 months, respectively), and the clinical benefit was not provided.

Capecitabine is a commonly used salvage regimen in anthracycline- and taxane-pretreated patients. Blum *et al* (2001) treated 162 patients who had received prior paclitaxel and anthracyclines with capecitabine. Median PFS, response duration, and OS were 3.1, 8.0, and 12.8 months, respectively. In a recent phase III study, Miller *et al* (2005) randomly assigned 462 women (15% previously untreated, 46% previously treated with one, and 34% with two chemotherapies) with MBC to receive capecitabine alone or in combination with Bevacizumab. The objective response rate in the capecitabine group was 9.1%. Median PFS, duration of response, and OS were 4.2, 7.6 (5 months for the combination group), and 14.5 months, respectively, and bevacizumab did not improve PFS or OS. Notably, a significant proportion (15%) of the patients in this study represented previously untreated women who were more likely to respond to treatment. The rate of taxane-pretreated patients in our study was slightly lower than in the later study (64 *vs* 85%), but our patients had received more previous therapies. Therefore, even though there are limitations to cross-study comparisons, one may conclude that the activity of PLD in previously treated patients with MBC seems to be similar to that of capecitabine. Generally, the efficacy of currently available drugs in this situation must be considered as low. The use of an ineffective drug for patients with previously treated MBC in two recent phase II studies resulted in a clinical benefit rate of 4.9 and 1.7%, respectively (Albain *et al*, 2002; Von Minckwitz *et al*, 2005). Therefore, the 24% clinical benefit rate observed here, although modest, can be related to the treatment with PLD. In our study, PLD was generally well tolerated and the safety profile observed here was consistent with that observed in earlier studies using PLD at 50 mg m^−2^ every 4 weeks (Nagore *et al*, 2000; O'Brien *et al*, 2004). No cardiac toxicities were seen and no significant changes in electrocardiograms or echocardiograms were observed during the study. Therefore, cardiac monitoring every other cycle may not be necessary, at least for patients receiving total doses of anthracyclines that are comparable to those applied in our study. In conclusion, our results indicate that the use of PLD is associated with an evident clinical benefit in patients previously treated with conventional anthracyclines. This clinical benefit is highest in patients with less exposure to prior chemotherapy regimens and taxane naive patients, and lowest in patients with documented anthracycline resistance.

## Figures and Tables

**Table 1:** Patient characteristics

**Patient characteristics**	**No. of patients (*n*=79)**	**%**
*Age (years)*		
Median	58	
Range	35–79	
		
*Karnofsky performance status* (%)		
100	18	22.7
90	27	34.1
⩽80	34	43.2
		
*No. of metastatic sites*		
1	26	32.9
2	25	31.6
⩾3	28	35.4
		
Oestrogen and/or progesterone receptor positive	68	86.0
HER2 positive^a^	4	5.1
		
*Site of disease*		
Bone only	5	6.3
Non-visceral soft tissue only	9	11.1
Visceral	65	82.6

a =3+ by immunohistochemistry or amplification by fluorescence *in situ* hybridization.

**Table 2:** Treatment history

	**No. of patients (*n*=79)**	**%**
Surgery	79	100.0
Adjuvant radiation therapy	58	73.4
Hormonal therapy^a^	68	86.0
Previous adjuvant chemotherapy	44	55.6
*Previous chemotherapy for MBC*		
1 regimen	30	37.9
2 regimens	23	29.1
⩾3 regimens	26	32.9
		
*Previous anthracycline-based chemotherapy*	79	100.0
Adjuvant only	18	22.8
Metastatic only	54	68.4
Both settings	7	8.9
		
*Anthracycline-free interval (months)*		
0–12	33	41.8
>12	46	58.2
		
*Dose of previous anthracycline-based chemotherapy* b		
Epirubicin (median, 360 mg m^−2^; range 140–810)	56	70.9
Mitoxantrone (median, 90 mg m^−2^; range 12–132)	11	13.9
Doxorubicin (median, 300 mg m^−2^; range 105–400)	8	10.1
		
*Previous taxane-based chemotherapy*	54	68.4
Adjuvant only	3	5.6^c^
Metastatic only	51	94.4^c^
Both settings	0	0
		
*Taxane-free interval (months)*		
0–12	39	72.2^c^
>12	15	27.8^c^

MBC=metastatic breast cancer.

a Adjuvant and/or metastatic.

b Six patients received an unknown cumulative dose of anthracyclines; two patients received epirubicin and mitoxantrone.

c In percentage of taxane-pretreated patients, *n*=54.

**Table 3:** Toxicities according to National Cancer Institute common toxicity criteria version 2.0

	**Grade 1–2 (*n*=79)**	**Grade 3–4 (*n*=79)**	**All grades (*n*=79)**
**Toxicities**	***n* (%)**	***n* (%)**	***n* (%)**
*Nonhaematologic*			
Alopecia	42 (53.8)^a^		42 (53.8)
Nausea	33 (42.3)	4 (5.1)	37 (47.4)
Diarrhoea	12 (15.3)	0	12 (15.3)
Vomiting	25 (32.0)	2 (2.5)	27 (34.6)
Constipation	24 (30.7)	3 (3.8)	27 (34.6)
Fever	15 (19.2)	0	15 (19.2)
Infection	16 (20.5)	6 (7.6)	22 (28.2)
Neurosensory	23 (29.4)	2 (2.5)	25 (32.0)
PPE	31 (39.7)	5 (6.4)	36 (46.1)
Mucositis	26 (33.3)	8 (10.2)	34 (43.5)
			
*Haematologic (n=76)*			
Neutropenia	25 (32.8)	13 (17.1)	38 (50.0)
Leukopenia	44 (57.8)	11 (14.4)	55 (72.3)
Anemia	60 (78.9)	7 (9.2)	67 (88.1)
Thrombocytopenia	23 (30.2)	3 (3.9)	26 (34.2)

PPE=palmar–plantar erythrodysesthesia

a Grade 1, 20; grade 2, 22; most events of alopecia were considered not related to PLD but to prior therapies by the investigator.

**Table 4:** The clinical benefit by the number of prior chemotherapies for MBC

	**Clinical benefit ⩾6 months^a^**	
**No. of previous chemotherapies for MBC**	***N***	**%**
All assessable pts, *n*=79	19	24.0
1 regimen, *n*=30 pts	9	30.0
2 regimens, *n*=23 pts	5	21.7
⩾3 regimens, *n*=26 pts	5	19.2

MBC=metastatic breast cancer; pts=patients.

a Defined as patients having an objective response of a stable disease of ⩾6 months duration.

**Table 5:** The clinical benefit by previous anthracycline and taxane treatment

	**Clinical benefit ⩾6 months^a^**	
**Number of previous chemotherapies for MBC**	***N***	**%**
No taxane, *n*=25 pts	10	40.0
Taxane, *n*=54 pts	9	16.6
Anthracycline-resistant, *n*=31 pts^b^	5	16.1
Non-anthracycline-resistant, *n*=48 pts	14	29.1
		
*Anthracycline-free interval* c		
0–12, *n*=29 pts	7	24.1
>12, *n*=32 pts	8	25.0

MBC=metastatic breast cancer; pts=patients.

a Defined as patients having an objective response or a stable disease of ⩾6 months duration.

b Defined as having disease progression on anthracycline-based therapy for MBC or within 6 months of adjuvant anthracycline-based therapy.

c Metastatic only.

## **Appendix**

### PARTICIPATING INSTITUTIONS AND INVESTIGATORS INCLUDE THE FOLLOWING

Klinik Bad Trissl, Oberaudorf, Germany (Klaus Gutschow, MD; Joachim Bischoff, MD); Krankenhaus Nordwest, Frankfurt, Germany (Elke Jäger, MD; Salah-Eddin Al-Batran, MD); Universitäts-Frauenklinik Frankfurt, Frankfurt, Germany (Manfred Kaufmann MD, Gunter von Minckwitz, MD); Hämatologisch-Onkologische Praxis Altona, Hamburg, Germany (Ulrich R. Kleeberg, MD); Krankenhaus Holweide, Köln, Germany (Ingo Meuthen, MD); Klinikum Berlin-Buch, Berlin, Germany (Günter Morack, MD); Katholisches Krankenhaus ‘St Nepomuk’, Erfurt, Germany (Christina Müller, MD); St Marien Krankenhaus Hamm, Hamm, Germany (Heinz Dürk, MD); St Vincentius-Klinik Karlsruhe, Karlsruhe, Germany (Hans-Gerd Meerpohl, MD); Universitätsklinikum Bonn, Bonn, Germany (Thomas Bauknecht, MD); Klinikum Chemnitz, Chemnitz, Germany (Klaus Renziehausen, MD); Onkologische Praxis, Ingolstadt, Germany (Peter Maubach, MD); Universitäts-Frauenklinik Tübingen, Tübingen, Germany (Diethelm Wallwiener, MD); Frauenklinik der Universität Ulm, Ulm, Germany (Rolf Kreienberg, MD); Dr-Horst-Schmidt-Kliniken, Wiesbaden, Germany (Andreas du Bois, MD); Klinikum St Marien, Amberg, Germany (Anton Scharl, MD); Universitätsklinikum Hamburg-Eppendorf, Hamburg, Germany (Fritz Jänicke, MD); Onkologische Praxis, Hamburg, Germany (Andreas Kopp, MD); Hämato-Onkologische Schwerpunktpraxis, Augsburg, Germany (Bernhard Heinrich, MD); Onkologische Gemeinschaftspraxis, Halle, Germany (Robert Rohrberg, MD); Martin-Luther-Universität Halle-Wittenberg, Halle, Germany (Heinz Kölbl, MD); Universitätsklinikum Homburg/Saar, Homburg/Saar, Germany (Werner Schmidt, MD); Städtische Kliniken Oldenburg, Oldenburg, Germany (Hans J. Illiger, MD); Klinikum Quedlinburg, Quedlinburg, Germany (Otto Boldt, MD); Ammerland Klinik, Westerstede, Germany (Karl Werner Schweppe, MD); Universitätsspital Zürich, Zürich, Switzerland (Alexander Knuth, MD).
